# A Robot Platform for Highly Efficient Pollutant Purification

**DOI:** 10.3389/fbioe.2022.903219

**Published:** 2022-06-17

**Authors:** Haocheng Wang, Shimin Yu, Junjie Liao, Xudong Qing, Daxing Sun, Fengtong Ji, Wenping Song, Lin Wang, Tianlong Li

**Affiliations:** ^1^ State Key Laboratory of Robotics and System, Harbin Institute of Technology, Harbin, China; ^2^ The Seventh Oil Production Plant of Changqing Oilfield Company, Xi’an, China; ^3^ Department of Mechanical and Automation Engineering, The Chinese University of Hong Kong, Hong Kong, China; ^4^ Chongqing Research Institute of HIT, Chongqing, China

**Keywords:** Janus microrobot, magnetic propulsion, flapping-wing micro air vehicle, oil adsorption, pollutant purification

## Abstract

In this study, we propose a highly efficient robot platform for pollutant adsorption. This robot system consists of a flapping-wing micro aircraft (FWMA) for long-distance transportation and delivery and cost-effective multifunctional Janus microrobots for pollutant purification. The flapping-wing micro air vehicle can hover for 11.3 km with a flapping frequency of approximately 15 Hz, fly forward up to 31.6 km/h, and drop microrobots to a targeted destination. The Janus microrobot, which is composed of a silica microsphere, nickel layer, and hydrophobic layer, is used to absorb the oil and process organic pollutants. These Janus microrobots can be propelled fast up to 9.6 body lengths per second, and on-demand speed regulation and remote navigation are manageable. These Janus microrobots can continuously carry oil droplets in aqueous environments under the control of a uniform rotating magnetic field. Because of the fluid dynamics induced by the Janus microrobots, a highly efficient removal of Rhodamine B is accomplished. This smart robot system may open a door for pollutant purification.

## Introduction

The concept of micro air vehicles was proposed by the American Defense Advanced Research Projects Agency in 1992. These vehicles were used to perform different types of tasks in complex environments. With the emergence of miniaturized aerial vehicles, some scholars subdivide micro air vehicles into the following branches: miniature unmanned air vehicle (miniature UAV), micro air vehicle (MAV), nano air vehicle (NAV), and pico air vehicle (PAV) (Hassanalian & Abdelkefi, 2017). MAV flight wings include fixed wing, rotary wing, rotor wing composite, or flapping wing. Compared with other types of wings, a flapping-wing micro air vehicle (FWMAV) imitates the flapping-wing flight modes of insects, birds, or bats. The FWMAV can take off and land vertically, hover in the air, and fly sideways and upside down. Some can also glide, fly at high speed, and cruise over long distances with low noise, high concealment, high flexibility, and little damage during contact with people (Hassanalian et al., 2015). The FWMAV provides a promising pathway for transporting large amounts of microrobots to the destination in the future.

Micro- and nanorobots have shown characteristic behaviors, such as geotaxis (Boiteau & MacKinley, 2014), chemotaxis (Mei et al., 2011; Jurado-Sanchez et al., 2015; Xu et al., 2015; Xing et al., 2019; Mou et al., 2021), phonotaxis (Ding et al., 2012; Wang et al., 2014; Melde et al., 2016), magnetotaxis (Peyer, Zhang, & Nelson, 2013; Li J. et al., 2016; Jurado-Sanchez et al., 2017; Jin et al., 2019; Xu et al., 2020), galvanotaxis (Klapper et al., 2010; Prusa & Cifra, 2019), phototaxis (Eelkema et al., 2006; Wu et al., 2014; Dai et al., 2016; Dai et al., 2016; Dong et al., 2016; Li T. et al., 2016; Mou et al., 2016; Zhou et al., 2018; Mou et al., 2019), and thermotaxis (Cai et al., 2017; Zhu et al., 2020). They have been widely used in targeted drug delivery, cell manipulation and separation, and environmental remediation (Jang et al., 2014; Dong et al., 2015; Ma et al., 2016; Wang et al., 2016; Chang et al., 2019a; Chang et al., 2019b; Ramos-Docampo et al., 2019; Wu et al., 2019; Yin et al., 2019; Xie et al., 2020). Because of their autonomous propulsion and versatile functions, magnetically propelled micromotors present great potential in aqueous environments (Li J. et al., 2016; Lin et al., 2018; Wang et al., 2018; Yu et al., 2019; Ji et al., 2021). Helical microswimmers (Wang et al., 2018; Jeon et al., 2019; Liu et al., 2021; Dong et al., 2022) and surface microrobots have achieved precise navigation in micropores and microchannels under the remote propelling and steering triggered by external magnetic fields (Li et al., 2017; Xie et al., 2019). In particular, magnetically propelled surface microrobots are capable of autonomously circumventing 3D barriers and overcoming complex cracks, opening new possibilities for a wide range of applications at the nanoscale (Li et al., 2018; Yu et al., 2019; Feng et al., 2021; Yu et al., 2021; Yu et al., 2022). These studies indicate that magnetically controlled Janus micro- and nanorobots are suitable for specific work in aqueous media.

In addition to controllable motion performance, the ability to adsorb oil droplets and organic pollutants can be achieved by surface modification, which is essential for pollutant purification (Zhao et al., 2011; Guix et al., 2012; Zhao & Pumera, 2014). Microrobots modified via surface functionalization adsorb the target substance through the bond link. For example, Gao et al. (2013) reported that a Janus motor consists of hydrophobic octadecyl-trichlorosilane–modified silica microspheres with a catalytic Pt hemisphere patch, which has diverse applications in aqueous environments. In addition, Soler et al. (2013)reported catalytically self-propelled microjets for degrading organic pollutants in water via Fenton oxidation. In follow-up studies, the positively and negatively charged and the neutral surfactant-infused polymer capsules all showed the capability of gathering oil droplets (Moo & Pumera, 2015). However, previous research is focused on structure optimization (e.g., metal−organic framework (Ying et al., 2019)) and functional modification of the surface (e.g., superhydrophobic) to enhance the adsorption capacity of oil and other pollutants. Moreover, some microrobots possess a shortened lifespan due to self-consumption during motion (Mou et al., 2015; Parmar et al., 2018; Ying & Pumera, 2019). Therefore, it is also of great importance to design a cost-effective robot system with the ability of long-distance transportation to improve the efficiency and accuracy of pollutant purification in water.

The objective of this work is to investigate a cost-effective robot system with the ability for long-distance transportation and delivery, and to create a highly efficient platform for pollutant adsorption. The motion ability of an FWMAV is investigated. Magnetically propelled multilayered polymetric microrobots are fabricated and characterized. The efficiency of self-propulsion and water decontamination is evaluated. This new multifunctional Janus microrobot is thus expected to improve the efficiency of pollutant purification in aqueous media.

## Materials and Methods

### Components of a Flapping-Wing Micro Air Vehicle

An FWMAV uses a small brushless motor (Lanxin 2807, China), a two-stage gear drive system, and two spatial 4-bar mechanisms to realize synchronous flapping of wings. One steering engine (Skye 14 mg, China) was used to change the wings’ angle of attack, and two steering engines were used to realize the pitching and rolling of the tail. The mass of an FWMAV is 430 g (without battery). The wingspan and wing chord are 1.1 and 0.2 m, respectively. The length of an FWMAV with the tail is 0.65 m. The length and width of the tail are 0.2 m.

### Fabrication of (PSS/PAH)_5_-Coated Janus Microrobots

The 5-, 8-, 10-, 12-, and 15-μm SiO_2_ microspheres were first washed with piranha solution. The microspheres were then placed on a glass slide and deposited in a 100-nm nickel layer using ion-sputtering equipment (K575XD, Emitech, England) at a 90° incident angle. After a brief sonication in ultrapure water, the Janus microspheres were released from the slide and dispersed in ultrapure water. The Janus microspheres were immersed in a 2-mg/ml PAH solution containing 0.5 M NaCl for 15 min with continuous shaking. The PAH-adsorbed Janus microspheres were centrifuged and washed three times using 0.1 M NaCl solution. The PAH-adsorbed Janus microspheres were then immersed in a 2-mg/ml PSS solution containing 0.5 M NaCl for 15 min with continuous shaking, followed by three-time repeated centrifugation/washing steps. The (PSS/PAH)_5_-coated Janus microspheres were obtained by repeating the aforementioned deposition procedure.

### Hydrophobic Surface Modification on Janus Microrobots

The (PSS/PAH)_5_-coated Janus microspheres were modified with the silane coupling agent, noctyltriethoxysilane. A combination of 1 ml coupling agent, 1 ml deionized (DI) water, 1 ml ammonium hydroxide (25 wt%), and 10 ml ethanol were mixed in a three-necked round-bottom flask by electrical stirring. The mixture was placed in a 50°C environment for 30 min to complete hydrolysis reaction. Afterward, the dehydrated microspheres were added to the mixture and stirred vigorously for 3 h. Finally, the modified microspheres were washed with ethanol three times and dried in a vacuum oven at 40°C for 12 h. Such functionalization involves the formation of a hydrophobic layer by self-assembly of long alkanethiol chains on the rough surfaces of microspheres.

### Characterization of Janus Microrobots

Scanning electron microscopy (SEM) analysis was performed with a Hitachi S-4300 instrument at an operating voltage of 10 keV. Mapping analysis was conducted by using an Oxford energy-dispersive X-ray spectroscope (EDX) attached to an SEM instrument and operated by Inca software. Videos of Janus microrobots were captured at 25 frame s^−1^ by using an inverted optical microscope (IX73, Olympus, Japan) coupled with a ×20 objective and a Point Grey CCD camera. The video data were analyzed using ImageJ and MATLAB to obtain the trajectories and velocities of the microrobots.

### Setup for Generating a Rotating Magnetic Field

The setup for generating an external rotating uniform magnetic field consists of Helmholtz coils with three degrees of freedom, a multifunction data acquisition, and a three single-channel output power amplifier. Based on controlling the current and voltage of the Helmholtz coils, an external rotating uniform magnetic field can be generated in any plane of the 3D space to actuate the microrobots in different motion modes. The external magnetic field setup was placed on the observation platform of the microscope to achieve real-time observation of the Janus microrobots.

## Results and Discussion

### Design and Preparation of the Smart Robot Platform

**FIGURE 1:**
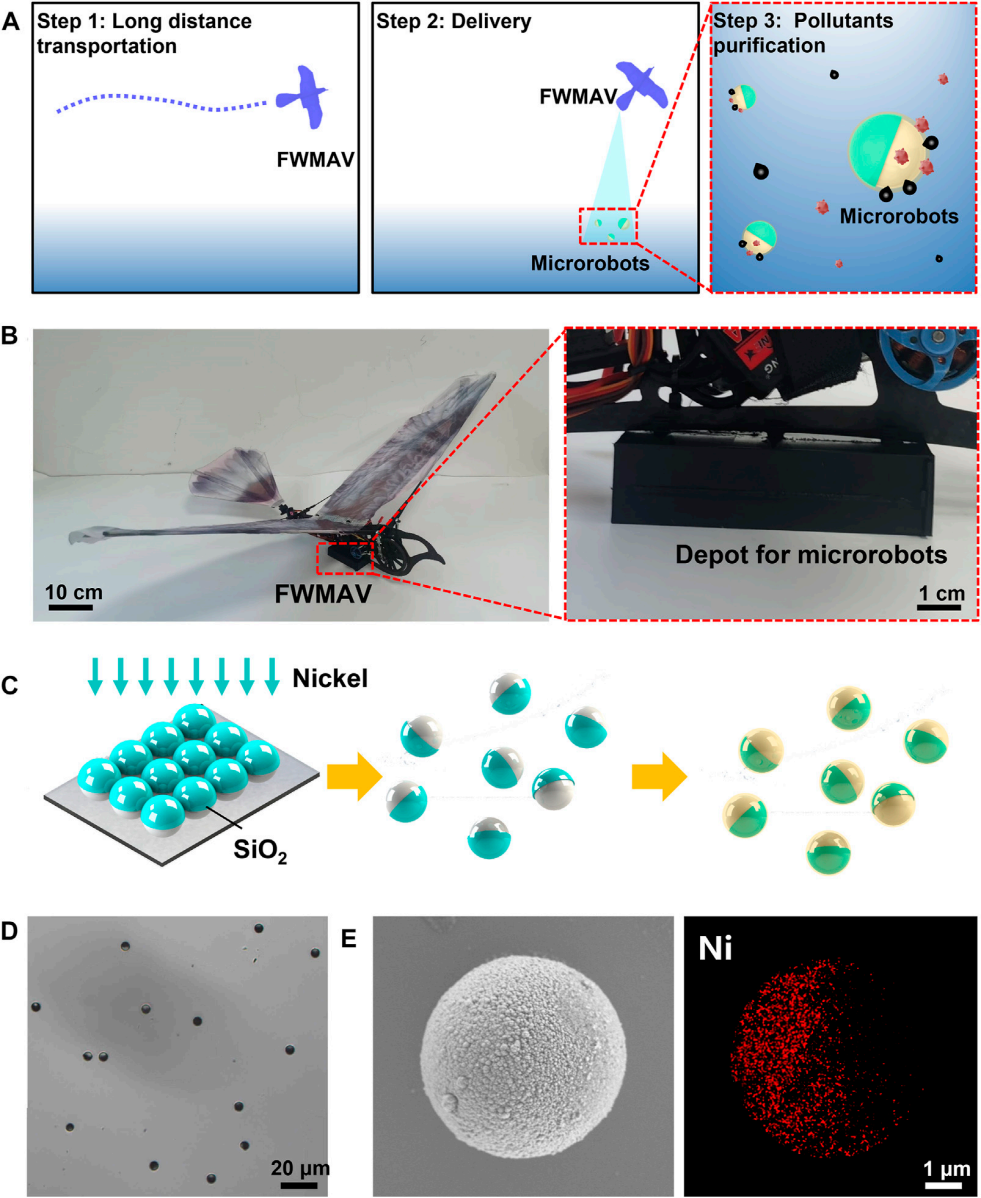
Design and preparation of the smart robot platform. **(A)** Schematic of the smart robot platform composed of an FWMAV and Janus microrobots. **(B)** Structure of an FWMAV. The depot for microrobots was fixed in the barycenter of the FWMAV. **(C)** Fabrication of Janus microrobots. **(D)** Optical microscopy image, **(E)** SEM image, and corresponding EDX mapping of oil-sorption Janus microrobots.

To create a highly efficient platform for pollutant adsorption, magnetically propelled Janus microrobots combined with an FWMAV were used to remove the residual oil and organic pollutants from aqueous media. As shown in Figure 1A, the FWMAV transports microrobots to the polluted environment, and then spray the microrobots by changing the angle of attack. The microrobots were composed of silica microspheres, a nickel layer polystyrene sulfonate (PSS)/polyallylamine hydrochloride (PAH) multilayer, and hydrophobic layer. The multilayered polymer was a cost-effective microscale or nanoscale adsorbent for the removal of organic pollutants in water. The hydrophobic layer was used to adsorb and carry the residual oil. Hence, the surface-modified magnetically propelled microrobots and FWMAV systems are essential to achieve this goal. The FWMAV has a length of 0.65 m and a width of 1.1 m, as shown in Figure 1B. The depot, which was used to store 80 g microrobots, was fixed in the barycenter of the FWMAV. The fabrication of Janus microstructures with five bilayers of PSS/PAH and modification of oil-sorption hydrophobic layers are presented in Figure 1C. These Janus microspheres were fabricated by half-coating silica microspheres with a thick nickel layer using electron beam evaporation. Five bilayers of PSS/PAH were deposited on the surfaces of Janus SiO_2_ microspheres via layer-by-layer self-assembly. Such functionalization involves the formation of a hydrophobic layer by self-assembly of long alkanethiol chains on the rough surfaces of microspheres. The surface morphology of these Janus microrobots with a diameter of 5 μm was characterized by optical microscopy, scanning electron microscopy, and energy-dispersive X-ray spectroscopy map analysis to examine the nickel composition of oil-sorption Janus microrobots (Figures 1D, E).

### Flying Performance of the Flapping-Wing Micro Air Vehicle

To investigate the mechanism of the FWMAV, a simple model was used to simulate the wind tunnel experiment simulation by XFlow. XFlow adopts the particle tracking method based on the three-dimensional Boltzmann model of particles, and the evolution equation is

$$f_{i}\left(x+e_{i}\delta_{i},t+\delta_{i}\right)-f_{i}\left(x,t\right)=\frac{1}{\tau }\left(f_{i}\left(x,t\right)-f_{i}^{eq}\left(x,t\right)\right),$$ (1)

$\text{where}\;f_{i}$ is the particle velocity distribution function, $\;f_{i}^{eq}$ is the local equilibrium function, τ is the relaxation time of dimension one, $\;e_{i}$ is the discrete velocity, and $\delta_{i}$ is the time step.

The equilibrium distribution function is

$$f_{i}^{eq}\left(x,t\right)=\rho w_{i}\left[1+\frac{e_{i}\cdot u}{c_{s}^{2}}+\frac{{\left(e_{i}\cdot u\right)}^{2}}{2c_{s}^{4}}-\frac{u^{2}}{2c_{s}^{2}}\right],$$ (2)

where $w_{i}$ is the weight coefficient and *c*_*s*_ is a parameter related to the speed of sound; the fluid macroscopic density *ρ* and velocity *u* can be shown as follows:

$$\rho =\sum_{i}f_{i}^{eq},$$ (3)

$$\rho u=\sum_{i}c_{i}f_{i}^{eq}.$$ (4)

The size of the wind tunnel is 5 m*2 m*2 m, and the incoming velocity is 5 m/s. The flapping frequency is 10 Hz. The angle of attack, the upward flapping amplitude, and the downward flapping amplitude are 25°, 45°, and 15°, respectively. The motion function is

$$\theta_{x}=30{}^{\circ}\cos\left(2\pi \mathrm{ft}\right)+15{}^{\circ}.$$ (5)

**FIGURE 2:**
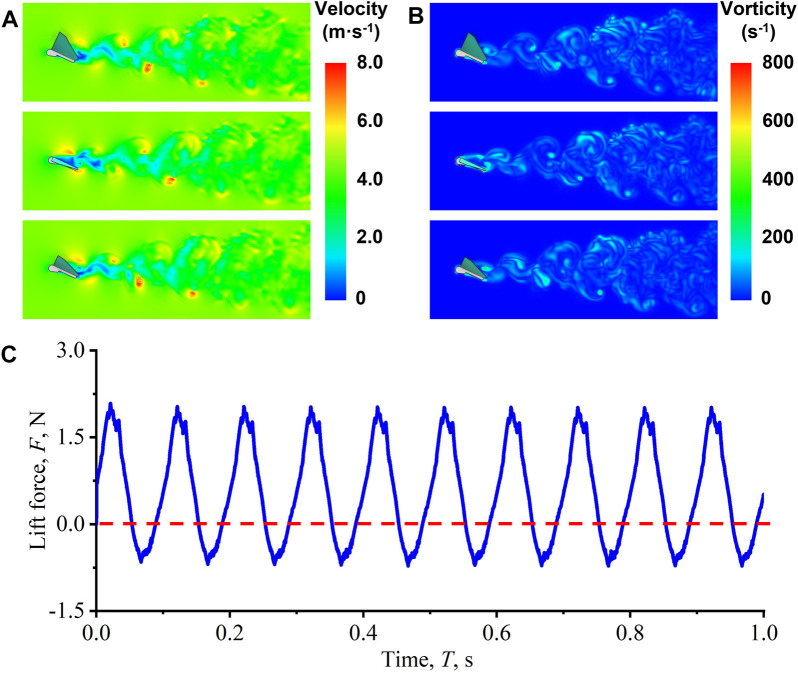
Characterization of the dynamic motion of the FWMAV. **(A)** Velocity contours of the FWMAV. **(B)** Vorticity contours of the FWMAV. **(C)** Simulated lift force of the FWMAV over 1 s.

The vorticity and velocity near the wings are shown in Figures 2A, B. Flapping wings can produce a series of Karman vortex streets on the trailing edge, thereby affecting the thrust of flight. Figure 2C presents the simulated lift force of the FWMAV over 1s. During the upward flapping of the wings, the lift force produces a trough, which is a negative lift. During the downward flapping of the wings, the lift force produces a wave crest, which is a positive lift. The flapping time difference improves the lift force. Because of the flapping motion of the wings, the FWMAV could achieve unique aerodynamic advantages over the traditional fixed wing or rotary flight when the feature size is on a small scale.

**FIGURE 3:**
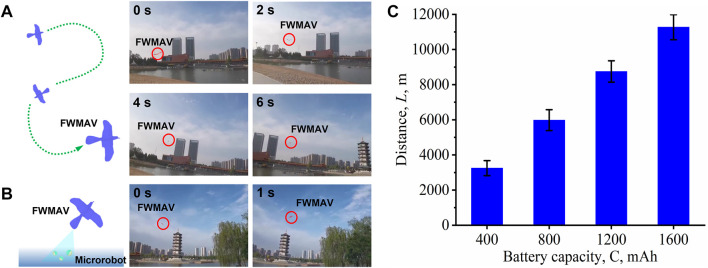
Flying performance of the FWMAV. **(A)** Schematic and time-lapse images depicting the efficient locomotion of the FWMAV. **(B)** Schematic and time-lapse images depicting the delivery of microrobots from the FWMAV. **(C)** Locomotion distances of the FWMAV with different battery capacities.

Figure 3A, along with the corresponding Supplementary Video S1, displays the continuous locomotion of the FWMAV in air. The FWMAV controls flight speed by changing the flapping frequency of the wings. The tail is used to control the pitch of the FWMAV by changing the angle of attack. If the FWMAV needs to yaw, it will roll the tail and lean the wings to one side at the same time. The maximum and minimum speeds of the FWMAV are 31.6 and 7.3 km/h, respectively. As shown in Figure 3B, as the FWMAV transports microrobots to the polluted environment, microrobots can be sprayed by changing the angle of attack. The effect of battery capacity on the locomotion distance of the FWMAV has also been investigated experimentally. As shown in Figure 3C, the locomotion distances of the FWMAV were 3.3, 6.0, 8.8, and 11.3 km with the battery capacities of 400, 800, 1,200, and 1,600 mAh, respectively. Because of its attractive performance, the FWMA can be used for long-distance transportation and delivery of microrobots.

**FIGURE 4:**
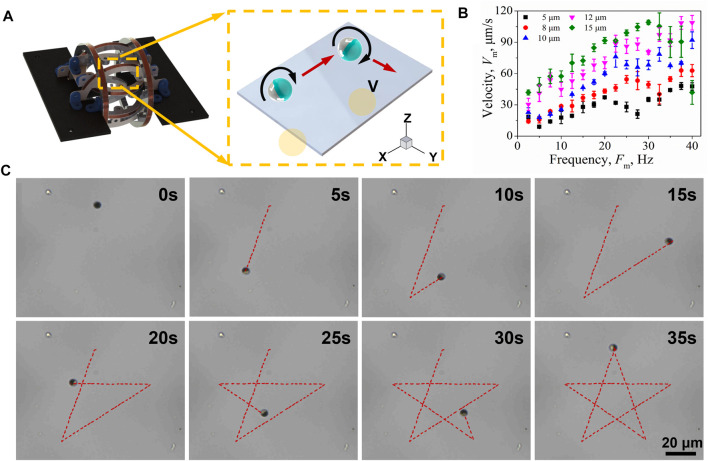
Propelling and steering of Janus microrobots. **(A)** Schematic of the uniform rotating magnetic field generation system. **(B)** Speed of Janus microrobots with different diameters of 5, 8, 10, 12, and 15 μm upon varying the magnetic frequency from 0 to 40 Hz. **(C)** Janus microrobots are steered along specific paths that form the “star” trajectory.

The abilities of remote and precise actuation are highly attractive features for microrobots in the application of environmental remediation. Here, we demonstrated the remote navigation strategy of Janus microrobots. Figure 4A shows a three-dimensional rotating magnetic field generator composed of three-degrees-of-freedom Helmholtz coils. The schematic of the controllable movement of a single Janus microrobot is presented in the inset. First, a circularly polarized rotating magnetic field is applied in the *x*-*z* plane and the microrobot is rolled along the *x* axis. The magnetic field is governed by

$$H\left(t\right)=H_{0}\left[\cos\left(\omega t\right)e_{x}+\sin\left(\omega t\right)e_{z}\right],$$ (6)

where *H*_0_ is the magnitude of *H*(*t*), *ω* is the angular frequency of the magnetic field, *t* is time, and **e**_*x*_ and **e**_*z*_ are the unit vectors along the *x* and *z* axes, respectively (hereafter, **e**_*y*_ is that along the *y* axis).

When the rotating magnetic field was changed and applied in the *y*-*z* plane, the direction of the microrobot motion changed to the *y* axis.

$$H\left(t\right)=H_{0}\left[-\cos\left(\omega t\right)e_{y}+\sin\left(\omega t\right)e_{z}\right].$$ (7)

The propulsion direction of the microrobot can be altered by changing the direction of the rotating magnetic field, which can be achieved by controlling the input current manually. It was essential to investigate the velocity of Janus microrobots since the efficiency of oil-sorption is affected by the motion of microrobots. When a microrobot was exposed to a rotating magnetic field, the torque induced by the rotating magnetic field and the viscous drag due to the surface caused it to roll forward along the surface. The dependence of the velocity of Janus microrobots with different sizes on the driving frequency was characterized, as shown in Figure 4B. The velocity of the 5-μm Janus motor increased from 9.0 to 48.1 μm/s (∼9.6 body length/s) upon increasing the driving frequency from 2.5 to 40.0 Hz with a magnetic strength of 20 mT. The 8-, 10-, and 12-μm Janus microspheres presented similar speed trends, and their speeds were high up to 71.8 μm/s (∼9.0 body length/s), 92.0 μm/s (∼9.2 body length/s), and 108.7 μm/s (∼9.1 body length/s), respectively. A linear relation is presented between the velocity of the Janus microsphere and driving frequency. The speeds of larger Janus microspheres are higher than those of smaller ones at the same driving frequency, while their relative speed is only frequency dependent. Notably, for 15-μm Janus microspheres, the speed increased linearly with the driving frequency and reached a maximum velocity of 109.0 μm/s (∼7.3 body length/s) at the frequency of 30 Hz and magnetic strength of 20 mT. Further increasing the frequency reduced the velocity. This maximum synchronization frequency is called the step-out frequency. We speculated that the reason for the decrease in speed is the occurrence of an out-of-step phenomenon and the increase in resistance caused by the increase in speed. The efficiency of pollutant purification is directly affected by the speed of the microrobot. Based on the results shown in Figure 4B, the 5-μm Janus microspheres were used as the preferred microrobots in the subsequent experiments. Controllable movement was the basis of the microrobot working in the micropores. Based on the aforementioned sensitive magnetic orientation, a microrobot walked along a predefined star-shaped trajectory as shown in Figure 4C (taken from Supplementary Video S2). The corners of the “star” track line were achieved by adjusting the angle of the magnetic field.

## Highly Efficient Pollutant Purification by Microrobots

**FIGURE 5:**
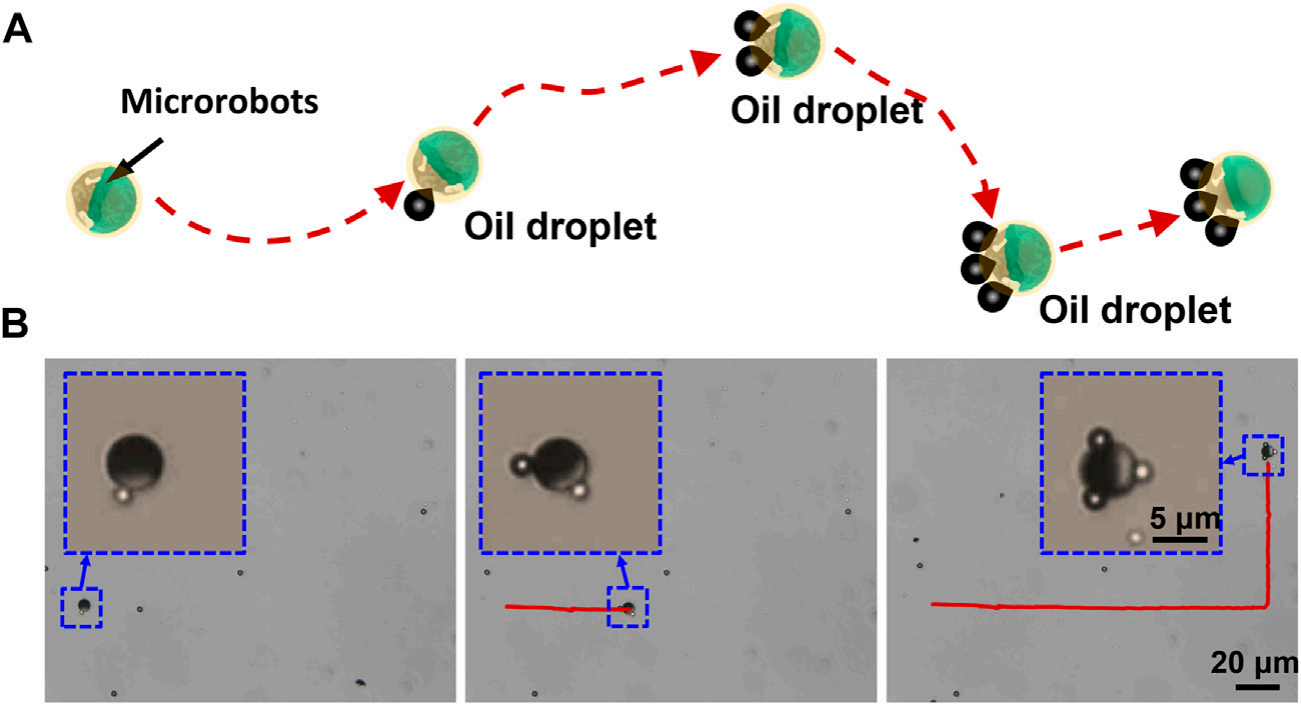
Picking up floating oil droplets using a Janus microrobot. **(A)** Schematic of a Janus microrobot continuously capturing oil droplets. **(B)** Time-lapse images of a Janus microrobot capturing several oil droplets randomly distributed in different positions.

Figure 5A demonstrates the oil-absorbing process of the microrobot in water. The microrobot sequentially approaches and captures multiple oil droplets, and finally transports the oil droplets in water. In our experiments, a 5-μm Janus microsphere served as the microrobot. A total of 20 μL crude oil was mixed with 1 ml deionized water. After ultrasonic treatment, the oil droplets were decomposed into small oil droplets with a diameter of about 1 μm, which were randomly distributed in the water. Figure 5B shows the process of a Janus microrobot adsorbing oil droplets via an external magnetic field with a frequency of 5 Hz over a period of 26 s (taken from Supplementary Video S3). Under the control of the uniform rotating magnetic field, the microrobot adsorbed three oil droplets in a sequence along the pre-programmed trajectory, and then carried and transported the oil droplets. It could be observed that the adsorbed oil droplets had little effect on the speed and trajectory of the microrobot, which was of great significance for actual oil removal.

**FIGURE 6:**
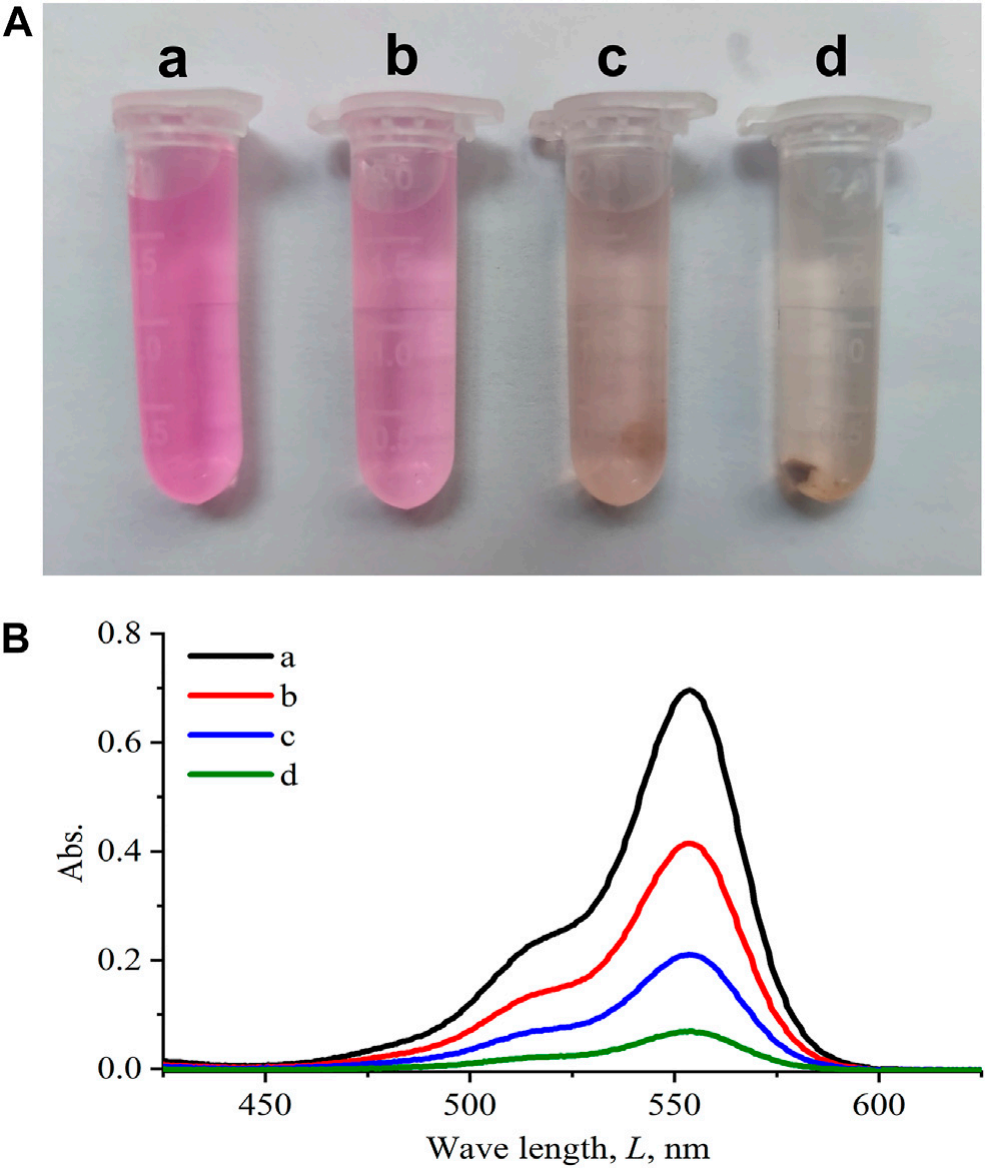
Removal of organic pollutants from aqueous solutions using Janus microrobots. **(A)** Photographs of a solution with 10 mg/L Rhodamine B after 5 min of treatment with equivalent amounts of pure water SiO_2_ **(a)**, SiO_2_ microspheres **(b)**, Janus microrobots without a magnetic field, and **(c)** Janus microrobots with a magnetic field **(d)**. **(B)** Absorbance spectra of Rhodamine B (*C*_*0*_ = 10 mg/L) after 5 min of treatment associated with Figure 6A.

Functionalization of (PSS/PAH)_5_ polymeric multilayers makes the microrobots physically absorb organic pollutants. To demonstrate the decontamination capability of the Janus microrobots, Rhodamine B was used as the target organic pollutant. The concentration of microrobots is about 500 per microliter. Experiments were performed in the following four combinations: in equivalent amounts of pure water (a) and SiO_2_ microspheres (b), the multilayered Janus microrobots without a magnetic field (c), and the multilayered Janus microrobots with a magnetic field (d). Figure 6A shows that the Janus microrobots were capable of absorbing Rhodamine B under propulsion in a magnetic field. As can be seen in Figure 6B, the peak absorption of Rhodamine B was approximately located at a wavelength of 554 nm. The absorption of Rhodamine B decreased significantly because of the treatment of Janus microrobots, that is, highly efficient removal of Rhodamine B was obtained because of the motion of the microrobots. The removal efficiency of Rhodamine B was about 90%. Because of the surface areas and selectively ionic interaction, the PSS/PAH multilayer showed a high absorbent capability of organic pollutants. In addition, the motion of the microrobots induced by the magnetic field enhanced the fluid dynamics and thus led to a high water purification efficiency.

## Conclusion

We have demonstrated a new highly efficient platform for pollutant adsorption that is composed of an FWMAV for long-distance transportation and delivery, and cost-effective multifunctional Janus microrobots for pollutant purification. The FWMAV can carry microrobots and fly to the designated location for 11.3 km with an approximate speed of 31.6 km/h. The Janus microrobots can be used for adsorbing the oil droplets and organic pollutants under the remote control of a uniform rotating magnetic field. The speed and the direction of a Janus microrobot with a diameter of 5 μm can be remotely modulated by controlling the magnetic field with a maximum speed of 48.1 μm/s, corresponding to a relative speed of ∼9.6 body lengths per second. The Janus microrobot can be propelled along the pre-programmable trajectory and sequentially adsorbs oil droplets that are randomly distributed in an aqueous environment. Highly efficient removal of Rhodamine B is obtained because of the active motion of the microrobots. Such a microrobot performs efficiently with autonomous motion, precise controllability, and the ability of pollutant purification, showing great potential in environmental remediation at the microscale.

## Data Availability

The original contributions presented in the study are included in the article/Supplementary Material; further inquiries can be directed to the corresponding authors.

## References

[B1] Boiteau G. MacKinley P. (2014). Geotaxis in the springtailFolsomia candida. Entomol. Exp. Appl. 152 (1), 16–22. 10.1111/eea.12195

[B2] Cai K. Yu J. Shi J. Qin Q.-H. (2017). Robust Rotation of Rotor in a Thermally Driven Nanomotor. Sci. Rep. 7, 46159. 10.1038/srep46159 28393898 PMC5385497

[B3] Chang X. Chen C. Li J. Lu X. Liang Y. Zhou D. (2019a). Motile Micropump Based on Synthetic Micromotors for Dynamic Micropatterning. ACS Appl. Mat. Interfaces 11 (31), 28507–28514. 10.1021/acsami.9b08159 31305060

[B4] Chang X. Tang W. Feng Y. Yu H. Wu Z. Xu T. (2019b). Coexisting Cooperative Cognitive Micro‐/Nanorobots. Chem. Asian J. 14 (14), 2357–2368. 10.1002/asia.201900286 30989807

[B5] Dai B. Wang J. Xiong Z. Zhan X. Dai W. Li C.-C. (2016). Programmable Artificial Phototactic Microswimmer. Nat. Nanotech 11 (12), 1087–1092. 10.1038/nnano.2016.187 27749832

[B6] Ding X. Lin S.-C. S. Kiraly B. Yue H. Li S. Chiang I.-K. (2012). On-chip Manipulation of Single Microparticles, Cells, and Organisms Using Surface Acoustic Waves. Proc. Natl. Acad. Sci. U.S.A. 109 (28), 11105–11109. 10.1073/pnas.1209288109 22733731 PMC3396524

[B7] Dong R. Li J. Rozen I. Ezhilan B. Xu T. Christianson C. (2015). Vapor-Driven Propulsion of Catalytic Micromotors. Sci. Rep. 5, 13226. 10.1038/srep13226 26285032 PMC4540091

[B8] Dong R. Zhang Q. Gao W. Pei A. Ren B. (2016). Highly Efficient Light-Driven TiO2-Au Janus Micromotors. ACS Nano 10 (1), 839–844. 10.1021/acsnano.5b05940 26592971

[B9] Dong Y. Wang L. Iacovacci V. Wang X. Zhang L. Nelson B. J. (2022). Magnetic Helical Micro-/nanomachines: Recent Progress and Perspective. Matter 5 (1), 77–109. 10.1016/j.matt.2021.10.010

[B10] Eelkema R. Pollard M. M. Vicario J. Katsonis N. Ramon B. S. Bastiaansen C. W. M. (2006). Nanomotor Rotates Microscale Objects. Nature 440, 163. 10.1038/440163a 16525460

[B11] Feng Y. Chang X. Liu H. Hu Y. Li T. Li L. (2021). Multi-response Biocompatible Janus Micromotor for Ultrasonic Imaging Contrast Enhancement. Appl. Mater. Today 23, 101026. 10.1016/j.apmt.2021.101026

[B12] Gao W. Pei A. Feng X. Hennessy C. Wang J. (2013). Organized Self-Assembly of Janus Micromotors with Hydrophobic Hemispheres. J. Am. Chem. Soc. 135 (3), 998–1001. 10.1021/ja311455k 23286304

[B13] Guix M. Orozco J. García M. Gao W. Sattayasamitsathit S. Merkoçi A. (2012). Superhydrophobic Alkanethiol-Coated Microsubmarines for Effective Removal of Oil. ACS Nano 6 (5), 4445–4451. 10.1021/nn301175b 22480219

[B14] Hassanalian M. Abdelkefi A. (2017). Classifications, Applications, and Design Challenges of Drones: A Review. Prog. Aerosp. Sci. 91, 99–131. 10.1016/j.paerosci.2017.04.003

[B15] Hassanalian M. Khaki H. Khosravi M. (2015). A New Method for Design of Fixed Wing Micro Air Vehicle. Proc. Institution Mech. Eng. Part G J. Aerosp. Eng. 229 (5), 837–850. 10.1177/0954410014540621

[B16] Jang S.-C. Hong S.-B. Yang H.-M. Lee K.-W. Moon J.-K. Seo B.-K. (2014). Removal of Radioactive Cesium Using Prussian Blue Magnetic Nanoparticles. Nanomaterials 4 (4), 894–901. 10.3390/nano4040894 28344255 PMC5308456

[B17] Jeon S. Kim S. Ha S. Lee S. Kim E. Kim S. Y. (2019). Magnetically Actuated Microrobots as a Platform for Stem Cell Transplantation. Sci. Robot. 4 (30), eaav431. 10.1126/scirobotics.aav4317 33137727

[B18] Ji F. Li T. Yu S. Wu Z. Zhang L. (2021). Propulsion Gait Analysis and Fluidic Trapping of Swinging Flexible Nanomotors. ACS Nano 15 (3), 5118–5128. 10.1021/acsnano.0c10269 33687190

[B19] Jin D. Yu J. Yuan K. Zhang L. (2019). Mimicking the Structure and Function of Ant Bridges in a Reconfigurable Microswarm for Electronic Applications. ACS Nano 13 (5), 5999–6007. 10.1021/acsnano.9b02139 31013052

[B20] Jurado-Sánchez B. Pacheco M. Rojo J. Escarpa A. (2017). Magnetocatalytic Graphene Quantum Dots Janus Micromotors for Bacterial Endotoxin Detection. Angew. Chem. Int. Ed. 56 (24), 6957–6961. 10.1002/anie.201701396 28504463

[B21] Jurado-Sánchez B. Sattayasamitsathit S. Gao W. Santos L. Fedorak Y. Singh V. V. (2015). Self-Propelled Activated Carbon Janus Micromotors for Efficient Water Purification. Small 11 (4), 499–506. 10.1002/smll.201402215 25207503

[B22] Klapper Y. Sinha N. Ng T. W. S. Lubrich D. (2010). A Rotational DNA Nanomotor Driven by an Externally Controlled Electric Field. Small 6 (1), 44–47. 10.1002/smll.200901106 19943245

[B23] Li J. Liu W. Li T. Rozen I. Zhao J. Bahari B. (2016). Swimming Microrobot Optical Nanoscopy. Nano Lett. 16 (10), 6604–6609. 10.1021/acs.nanolett.6b03303 27608508

[B24] Li T. Chang X. Wu Z. Li J. Shao G. Deng X. (2017). Autonomous Collision-free Navigation of Microvehicles in Complex and Dynamically Changing Environments. ACS Nano 11 (9), 9268–9275. 10.1021/acsnano.7b04525 28803481

[B25] Li T. Li J. Zhang H. Chang X. Song W. Hu Y. (2016). Magnetically Propelled Fish-like Nanoswimmers. Small 12 (44), 6098–6105. 10.1002/smll.201601846 27600373

[B26] Li T. Zhang A. Shao G. Wei M. Guo B. Zhang G. (2018). Janus Microdimer Surface Walkers Propelled by Oscillating Magnetic Fields. Adv. Funct. Mat. 28 (25), 1706066. 10.1002/adfm.201706066

[B27] Lin Z. Fan X. Sun M. Gao C. He Q. Xie H. (2018). Magnetically Actuated Peanut Colloid Motors for Cell Manipulation and Patterning. ACS Nano 12 (3), 2539–2545. 10.1021/acsnano.7b08344 29443501

[B28] Liu J. Yu S. Xu B. Tian Z. Zhang H. Liu K. (2021). Magnetically Propelled Soft Microrobot Navigating through Constricted Microchannels. Appl. Mater. Today 25, 101237. 10.1016/j.apmt.2021.101237

[B29] Ma X. Wang X. Hahn K. Sánchez S. (2016). Motion Control of Urea-Powered Biocompatible Hollow Microcapsules. ACS Nano 10 (3), 3597–3605. 10.1021/acsnano.5b08067 26863183

[B30] Mei Y. Solovev A. A. Sanchez S. Schmidt O. G. (2011). Rolled-up Nanotech on Polymers: from Basic Perception to Self-Propelled Catalytic Microengines. Chem. Soc. Rev. 40 (5), 2109. 10.1039/c0cs00078g 21340080

[B31] Melde K. Mark A. G. Qiu T. Fischer P. (2016). Holograms for Acoustics. Nature 537, 518–522. 10.1038/nature19755 27652563

[B32] Moo J. G. S. Pumera M. (2015). Chemical Energy Powered Nano/Micro/Macromotors and the Environment. Chem. Eur. J. 21 (1), 58–72. 10.1002/chem.201405011 25410790

[B33] Mou F. Xie Q. Liu J. Che S. Bahmane L. You M. (2021). ZnO-based Micromotors Fueled by CO2: the First Example of Self-Reorientation-Induced Biomimetic Chemotaxis. Natl. Sci. Rev. 8 (11), nwab066. 10.1093/nsr/nwab066 34876993 PMC8645024

[B34] Mou F. Kong L. Chen C. Chen Z. Xu L. Guan J. (2016). Light-controlled Propulsion, Aggregation and Separation of Water-Fuelled TiO2/Pt Janus Submicromotors and Their "On-The-Fly" Photocatalytic Activities. Nanoscale 8 (9), 4976–4983. 10.1039/c5nr06774j 26579705

[B35] Mou F. Pan D. Chen C. Gao Y. Xu L. Guan J. (2015). Magnetically Modulated Pot-like MnFe2O4Micromotors: Nanoparticle Assembly Fabrication and Their Capability for Direct Oil Removal. Adv. Funct. Mat. 25 (39), 6173–6181. 10.1002/adfm.201502835

[B36] Mou F. Zhang J. Wu Z. Du S. Zhang Z. Xu L. (2019). Phototactic Flocking of Photochemical Micromotors. Iscience 19, 415–424. 10.1016/j.isci.2019.07.050 31421596 PMC6704395

[B37] Parmar J. Vilela D. Villa K. Wang J. Sánchez S. (2018). Micro- and Nanomotors as Active Environmental Microcleaners and Sensors. J. Am. Chem. Soc. 140 (30), 9317–9331. 10.1021/jacs.8b05762 29969903

[B38] Peyer K. E. Zhang L. Nelson B. J. (2013). Bio-inspired Magnetic Swimming Microrobots for Biomedical Applications. Nanoscale 5 (4), 1259–1272. 10.1039/c2nr32554c 23165991

[B39] Prusa J. Cifra M. (2019). Molecular Dynamics Simulation of the Nanosecond Pulsed Electric Field Effect on Kinesin Nanomotor. Sci. Rep. 9, 19721. 31873109 10.1038/s41598-019-56052-3 PMC6928163

[B40] Ramos-Docampo M. A. Fernández-Medina M. Taipaleenmäki E. Hovorka O. Salgueiriño V. Städler B. (2019). Microswimmers with Heat Delivery Capacity for 3D Cell Spheroid Penetration. ACS Nano 13 (10), 12192–12205. 10.1021/acsnano.9b06869 31502822

[B41] Soler L. Magdanz V. Fomin V. M. Sanchez S. Schmidt O. G. (2013). Self-Propelled Micromotors for Cleaning Polluted Water. ACS Nano 7 (11), 9611–9620. 10.1021/nn405075d 24180623 PMC3872448

[B42] Wang W. Li S. Mair L. Ahmed S. Huang T. J. Mallouk T. E. (2014). Acoustic Propulsion of Nanorod Motors inside Living Cells. Angew. Chem. Int. Ed. 53 (12), 3201–3204. 10.1002/anie.201309629 PMC4069361 24677393

[B43] Wang X. Hu C. Schurz L. De Marco C. Chen X. Pané S. (2018). Surface-Chemistry-Mediated Control of Individual Magnetic Helical Microswimmers in a Swarm. ACS Nano 12 (6), 6210–6217. 10.1021/acsnano.8b02907 29799724

[B44] Wang Y. Li P. Truong-Dinh Tran T. Zhang J. Kong L. (2016). Manufacturing Techniques and Surface Engineering of Polymer Based Nanoparticles for Targeted Drug Delivery to Cancer. Nanomaterials 6 (2), 26. 10.3390/nano6020026 PMC5302480 28344283

[B45] Wu Z. Li L. Yang Y. Hu P. Li Y. Yang S. Y. (2019). A Microrobotic System Guided by Photoacoustic Computed Tomography for Targeted Navigation in Intestines *In Vivo* . Sci. Robot. 4 (32), aax0613. 10.1126/scirobotics.aax0613 PMC7337196 32632399

[B46] Wu Z. Lin X. Wu Y. Si T. Sun J. He Q. (2014). Near-Infrared Light-Triggered "On/Off" Motion of Polymer Multilayer Rockets. ACS Nano 8 (6), 6097–6105. 10.1021/nn501407r 24806430

[B47] Xie H. Sun M. Fan X. Lin Z. Chen W. Wang L. (2019). Reconfigurable Magnetic Microrobot Swarm: Multimode Transformation, Locomotion, and Manipulation. Sci. Robot. 4 (28), aav8006. 10.1126/scirobotics.aav8006 33137748

[B48] Xie H. Meng X. Zhang H. Sun L. (2020). Development of a Magnetically Driven Microgripper for PicoNewton Force-Controlled Microscale Manipulation and Characterization. IEEE Trans. Ind. Electron. 67 (3), 2065–2075. 10.1109/tie.2019.2905805

[B49] Xing Y. Zhou M. Du X. Li X. Li J. Xu T. (2019). Hollow Mesoporous carbon@Pt Janus Nanomotors with Dual Response of H2O2 and Near-Infrared Light for Active Cargo Delivery. Appl. Mater. Today 17, 85–91. 10.1016/j.apmt.2019.07.017

[B50] Xu T. Guan Y. Liu J. Wu X. (2020). Image-Based Visual Servoing of Helical Microswimmers for Planar Path Following. IEEE Trans. Autom. Sci. Eng. 17 (1), 325–333. 10.1109/tase.2019.2911985

[B51] Xu T. Soto F. Gao W. Dong R. Garcia-Gradilla V. Magaña E. (2015). Reversible Swarming and Separation of Self-Propelled Chemically Powered Nanomotors under Acoustic Fields. J. Am. Chem. Soc. 137 (6), 2163–2166. 10.1021/ja511012v 25634724

[B52] Yin T. Yang Z. Dong Z. Lin M. Zhang J. (2019). Physicochemical Properties and Potential Applications of Silica-Based Amphiphilic Janus Nanosheets for Enhanced Oil Recovery. Fuel 237, 344–351. 10.1016/j.fuel.2018.10.028

[B53] Ying Y. Pourrahimi A. M. Sofer Z. Matějková S. Pumera M. (2019). Radioactive Uranium Preconcentration via Self-Propelled Autonomous Microrobots Based on Metal-Organic Frameworks. ACS Nano 13 (10), 11477–11487. 10.1021/acsnano.9b04960 31592633

[B54] Ying Y. Pumera M. (2019). Micro/Nanomotors for Water Purification. Chem. Eur. J. 25 (1), 106–121. 10.1002/chem.201804189 30306655

[B55] Yu S. Li T. Ji F. Zhao S. Liu K. Zhang Z. (2022). Trimer-like Microrobots with Multimodal Locomotion and Reconfigurable Capabilities. Mater. Today Adv. 14, 100231. 10.1016/j.mtadv.2022.100231

[B56] Yu S. Ma N. Yu H. Sun H. Chang X. Wu Z. (2019). Self-Propelled Janus Microdimer Swimmers under a Rotating Magnetic Field. Nanomaterials 9 (12), 1672. 10.3390/nano9121672 PMC6956008 31771115

[B57] Yu S. Sun Z. Zhang Z. Sun H. Liu L. Wang W. (2021). Magnetic Microdimer as Mobile Meter for Measuring Plasma Glucose and Lipids. Front. Bioeng. Biotechnol. 9, 779632. 10.3389/fbioe.2021.779632 34900967 PMC8660689

[B58] Zhao G. Pumera M. (2014). Marangoni Self-Propelled Capsules in a Maze: Pollutants 'sense and Act' in Complex Channel Environments. Lab. Chip 14 (15), 2818–2823. 10.1039/c4lc00431k 24903774

[B59] Zhao G. Seah T. H. Pumera M. (2011). External-Energy-Independent Polymer Capsule Motors and Their Cooperative Behaviors. Chem. Eur. J. 17 (43), 12020–12026. 10.1002/chem.201101450 21953585

[B60] Zhou D. Gao Y. Yang J. Li Y. C. Shao G. Zhang G. (2018). Light-Ultrasound Driven Collective "Firework" Behavior of Nanomotors. Adv. Sci. 5 (7), 1800122. 10.1002/advs.201800122 PMC6051403 30027044

[B61] Zhu F. Guo Z. Chang T. (2020). Nanoscale Continuous Cyclic Motion Driven by a Stable Thermal Field. Appl. Mater. Today 18, 100520. 10.1016/j.apmt.2019.100520

